# Feeling Deficient but Reluctant to Improve: How Perceived Control Affects Consumers' Willingness to Purchase Self-Improvement Products Under Self-Deficit Situations

**DOI:** 10.3389/fpsyg.2021.544523

**Published:** 2021-05-28

**Authors:** Wei Song, Xiaotong Jin, Jian Gao, Taiyang Zhao

**Affiliations:** ^1^Department of Marketing, Business School, Jilin University, Changchun, China; ^2^Department of Marketing, School of Business and Administration, Zhejiang University of Finance and Economics, Hangzhou, China; ^3^Department of Psychology, School of Philosophy and Sociology, Jilin University, Changchun, China

**Keywords:** self-deficit, perceived control, locus of control, defense mechanism, self-improvement products

## Abstract

This study explored how perceived control affects consumers' willingness to purchase self-improvement products (WSP) under self-deficit situations. For this purpose, three experiments were conducted to examine the following sources of control: the controllability of self-deficits (Experiment 1); the locus of control (Experiment 2); and situational perceived control (Experiment 3). According to the results, higher perceived control can reduce consumers' defensive reaction tendencies, thus increasing their willingness to purchase products that claim to improve their current deficits. Moreover, the aforementioned effect only occurs in within-domain improvement products, rather than without-domain improvement products.

## Introduction

In daily life, individuals often encounter various events that make them feel deficient such as failing an examination, being criticized by others, facing a difficult challenge, etc. Since these events can cause them to feel psychologically threatened, they may become the stimuli that prompt them to consume certain products (Mandel et al., 2017). Among the many products in the market that can help consumers cope with such situations, self-improvement products are the most productive for enhancing their corresponding abilities and compensating for their deficiencies (Kim and Gal, 2014). Although self-improvement includes obvious advantages, some individuals may exhibit defensive behaviors toward any information that suggests that they are deficient in certain aspects (Ruttan and Nordgren, 2016). Consequently, they may become reluctant to adopt such methods altogether. For example, a student who has just failed an examination may become reluctant to engage in learning, while an obese individual may become reluctant to lose weight. In such situations, people may show a low *willingness to buy self-improvement products* (WSP). However, previous studies have paid little attention to such phenomena, and their underlying psychological mechanisms have yet to be revealed.

The research on self-deficits in consumer behavior has mainly focused on compensatory consumption and found that consumers may compensate for their self-deficit by purchasing a certain product (Kim and Gal, 2014; Mandel et al., 2017). However, as mentioned above, in some cases, if there is a self-deficit, consumers' willingness to buy self-improvement products may decrease. For example, Kim and Rucker (2012) found that after having suffered a certain self-threat, consumers are more likely to buy products that help distract them from threatening situations than products related to the threat. Such contradictions in literature may imply that in self-deficit situations, some consumers may have a lower WSP than others. However, the time when consumers' WSP increases or decreases has not been explored sufficiently.

Perceived control refers to individuals' cognition and feeling that they can control external factors and their environment. Much evidence has been presented that perceived control affects individuals' behaviors when they are in a self-deficit situation. A study found that individuals with varying levels of perceived control use different coping strategies to manage external threats (Rothbaum et al., 1982). When individuals have high perceived control over a difficult situation, they tend to solve the problem, and when they have low perceived control, they tend to try to escape from the problem (Zhao et al., 2020). However, the literature has mainly focused on social psychology, and empirical support for consumer behavior, especially purchasing behavior, remains insufficient. Self-deficit can be viewed as a threat, and purchasing self-improvement products can be viewed as an action to solve the problem. Building on this idea, we posit that perceived control may also affect consumers' WSP. Therefore, the first research objective of this article is to examine whether perceived control affects consumers' WSP under self-deficit situations.

The second research objective is to understand what mechanism and what types of influence paths of perceived control affect WSP. With regard to the underlying mechanism, the literature has mainly employed symbolic self-completion theory to explain why consumers use compensatory consumption to cope with self-deficit (Mandel et al., 2017). However, this theory explains why self-deficit leads consumers to buy a certain product but not why self-deficit makes consumers unwilling to buy it. Therefore, we aim to introduce a theory that explains why consumers show such a de-consumption tendency under self-deficit. The defense mechanism is a well-studied theory in social psychology mainly used to explain why individuals show defensive reactions (e.g., denial and isolation) when experiencing external threats (Baumeister et al., 1998). We argue that perceived control affects consumers' tendency for defensive reactions and that when consumers show a defensive reaction tendency to self-deficit, they are more likely to try to escape from problems than solve them by purchasing self-improvement products. Thus, in this study, we examine whether the defense mechanism theory suitably explains how the effects of perceived control on WSP are mediated. Our study contributes to the extant literature by introducing a new theory to the area of consumer behavior.

The literature on consumer behavior shows that consumers under threat may demonstrate distinct attitudes toward different categories of products (Mandel et al., 2017). Thus, product type might also be a crucial moderating variable in this study. The third research objective is to understand whether consumers show a low WSP for all types of products when there is a self-deficit. To achieve this objective, we explore the boundary condition from the perspective of product type. The literature has tended to distinguish compensatory consumption into within-domain compensation and without-domain compensation (Kim and Rucker, 2012; Mandel et al., 2017). Following this tradition, we divide self-improvement products into within-domain and without-domain products and then introduce product type as a moderator to explore whether the effect of perceived control on consumers' WSP is suitable for all types of products.

## Literature Review and Hypotheses Development

### Self-Deficit and WSP

According to the self-discrepancy theory, when consumers receive information indicating that they are deficient in certain aspects, they tend to feel discrepancies between their actual self and their ideal self (Higgins, 1987). This type of self-discrepancy can cause individuals to feel psychologically threatened, thus generating a strong motivation to eliminate this aversive state. Since using certain products can help consumers eliminate such a state, self-deficits become an important factor in promoting consumers to purchase such products (Mandel et al., 2017). The same study used the term *compensatory consumption behavior* to indicate any purchase, use, or consumption of products or services motivated by the desire to offset or reduce a self-discrepancy. Moreover, the object of compensatory consumption generally includes a wide range of products such as symbolic and hedonic ones (Rucker and Galinsky, 2008; Kim and Rucker, 2012).

Among the alternative choices of compensatory consumption, purchasing self-improvement products is considered the most productive approach for dealing with self-deficit situations (Han et al., 2015; Mandel et al., 2017). It has also become a common marketing strategy for advertisers in which their goal is to make consumers feel deficient in some aspect (Groesz et al., 2002). For example, an advertisement featuring a model with an ideal figure may make consumers feel that they are not attractive enough or a marketing strategy adopted by a health product manufacturer may make consumers feel inadequate in terms of their fitness. In such cases, the underlying logic is that when consumers feel inadequate, they will attempt to solve the problem by purchasing products that claim to reduce or even eradicate their deficiency.

Despite using self-improvement products to cope with self-deficit situations is often one of consumers' choice, the existing literature may imply that consumers are also often reluctant to choose this approach. For example, they may choose symbolic products that help them deny the deficiency by signaling that they are actually “masters” of the threatened domain (Kim and Gal, 2014), or they may choose hedonic products to help adjust their negative mood (Kim and Rucker, 2012), rather than improving themselves by using self-improvement products.

In other words, consumers, in some situations, may be more willing to buy self-improvement products to compensate for self-deficits; in other situations, they may also demonstrate a low WSP. However, the compensatory consumption literature has mainly focused on the former (Mandel et al., 2017) and ignored the latter. Of great value would be to study why consumers' WSP is low in a self-deficit situation. First, using self-improvement products is a productive method to help consumers manage self-deficits. If consumers show a low WSP, they will miss the opportunity for self-growth. Second, a common marketing strategy for businesses to promote consumers' WSP is manifesting their self-deficits. Thus, if practitioners do not realize the underlying psychological mechanism of such de-consumption behavior, their marketing strategies may conflict with their desires. Thus, examining the underlying psychological mechanisms that reduce consumers' WSP is a research objective of this study. The results of this examination can guide marketing strategies and contribute to the literature on this theoretical topic.

### Why Do Consumers Show a Low WSP in Self-Deficit Situations?

Theories on compensatory consumption have mainly been used to explain why self-deficits improve consumers' willingness to buy certain products; few theories have explained their unwillingness to do the same (Mandel et al., 2017). Therefore, this study discusses defense mechanism theory, originally a concept in social psychology, to explain why consumers show a low WSP in self-deficit situations.

In the field of social psychology, it has been found that when faced with stress and anxiety from external threats, individuals may employ psychological defense mechanisms to protect themselves, in addition to directly solving the problems (Baumeister et al., 1998). In this case, the term *defense mechanisms* refer to “the cognitive and behavioral tendencies that individuals unconsciously adopt in the face of setbacks or conflicts in order to relieve the resulted tension and anxiety, most of which are characterized by distorted cognition or self-deception” (Cramer, 2012). However, such mechanisms do not eliminate the source of the threat, but they simply change how individuals process information to reduce the impact of the self-threat.

Consumers adopting psychological defense mechanisms also exhibit defensive reactions to external threats. For example, Barkow (1989) found that individuals selectively ignore information that indicates that they are of low status while focusing more on information that makes them feel that they are of high status. In a related study, Crocker et al. (1991) found that when African-Americans receive negative evaluations from white people, they tend to attribute such evaluations to racial discrimination, as a means of denying their deficits. Similarly, Ditto and Lopez (1992) found that when individuals do not perform well on a test, they generally question the validity of the test and the accuracy of the results. In addition to denial, individuals may avoid the influence of psychological threats by isolating themselves from the sources, thoughts, and feelings of self-threats (Campbell and Sedikides, 1999). For instance, students who fail an examination may refuse to talk about it with others or they may avoid certain individuals or environments that remind them of the examination. Although the explanatory power of defense mechanism theory in individuals' social behavior has been proved, its role in consumer behavior remains unclear. To compensate for this gap, we propose the following logic to explain how defense mechanisms affect consumers' WSP.

Based on the fact that consumers purchase self-improvement products suggests that they recognize and accept their deficiencies, and aim to improve their corresponding abilities through such products (Kim and Gal, 2014). However, since consumers with defensive reaction tendencies may not think that they are deficient in any way, they may deem such products unnecessary. These products may also become a constant reminder of their deficits (Lisjak et al., 2015). For example, when a student with poor academic performance sees a book, he/she may feel a sense of powerlessness in learning. In this case, the book serves as a reminder of his/her deficiency in learning, thus generating resistance to the book itself. Moreover, some individuals may attack the object that makes them feel inadequate. For instance, Wan et al. (2009) found that individuals may criticize individuals who are considered “good-looking,” since they make them feel inferior in terms of attractiveness. Based on this logic, when consumers react defensively to psychological threats, they may be less willing to purchase products that can enhance their corresponding deficiencies. Since defense mechanisms can lead to the above unproductive results, it is important to explore the factors that can reduce such mechanisms and enhance consumers' WSP.

### Role of Perceived Control

According to the aforementioned rationale, consumers may show a low WSP when self-deficit information activates their defensive reaction tendency. By contrast, some studies have implied that self-deficit may also improve consumers' WSP (Mandel et al., 2017). Thus, finding a variable to reconcile the aforementioned contradictions is of substantial significance. A high and a low WSP may be the consequence of consumers using different strategies to manage self-deficits. Studies have implied that perceived control can affect individuals' choice of coping strategies to manage external threats (Zhao et al., 2020). Thus, we introduce perceived control as our dependent variable to examine when consumers show a low WSP under self-deficit situations and when they show a high WSP.

Perceived control refers to individuals' cognition and feeling that they can control external factors and their environment, which differs from the objective capacity of control that they possess. According to the two-process model of control (Rothbaum et al., 1982), individuals' control over their external environment is divided into two processes: primary control and secondary control. The former refers to individuals' efforts to change their environment or eliminate the sources of pressure to satisfy their needs and expectations. The latter refers to the condition in which individuals adjust themselves to the environment since they cannot change it or eliminate the sources of the pressure.

In general, when under stress, individuals tend to choose primary control as a means of dealing with the external environment. However, when their perceived control is low and they realize that they cannot solve their problems, they choose secondary control. In this case, although secondary control cannot directly solve their problems, it can reduce the impact of the stressful situation (Heckhausen and Schulz, 1995). Based on this logic, when individuals believe that they can control the situation in which they find themselves, they are likely to choose coping strategies such as self-improvement products to deal with their problems. Conversely, when individuals believe that they are unable to control the situation, they are more likely to adopt defense mechanisms to protect themselves, thus reducing their WSP.

According to the aforementioned rationale, we develop our hypotheses as follows: Because it takes time, effort, and money to improve certain deficiencies, individuals will consider and judge whether to adopt such a coping strategy or to choose to escape. Zhao et al. (2020) found that when individuals perceive that they can control the threats, they tend to adopt problem-focused coping strategies to solve the problem. Otherwise, they adopt emotion-focused coping strategies to regulate their negative mood. Consequently, when consumers perceive that their current self-deficits are controllable, they tend to compensate for the deficiencies and gain self-growth by purchasing self-improvement products. When they perceive self-deficits as uncontrollable, they tend to feel that it is futile to spend time, effort, or money to improve their corresponding deficiencies and show a low WSP. Thus, the following hypothesis is posited:

**Hypothesis 1:** Perceived control positively affects consumers' WSP under a self-deficit situation. More specifically, when consumers have low perceived control over the self-deficits, they will show low WSP; when they have high perceived control over self-deficits, they will show high WSP.

As for the underlying psychological mechanisms, consumers' defensive reaction tendencies mediate the aforementioned effect. More specifically, when individuals perceive their self-deficits are uncontrollable, they will turn to alternative measures to reduce their negative feelings. Since defense mechanisms can function as a buffer to alleviate the anxiety from psychological threats (Baumeister et al., 1998), individuals may exhibit higher defensive reaction tendencies toward the self-deficits that they believe are uncontrollable. For example, they may deny the existence of their deficiencies or isolate themselves from the related products that may remind them of such deficiencies (Campbell and Sedikides, 1999; Lin et al., 2003). In such situations, they will consider the self-improvement products useless or even avoid them altogether. Hence, the following hypothesis is posited:

**Hypothesis 2:** Defensive reaction tendencies mediate the effect of perceived control on WSP. More specifically, when consumers have low perceived control over self-deficits, they show stronger defensive reaction tendencies, which lower their WSP.

It is worth noting that perceived control can be obtained from multiple sources. First, as for the characteristic of deficiencies, individuals feel various types in their daily lives, some of which make them feel that they are controllable, and others that do not (Zhao et al., 2020). As elaborated above, this source of perceived control may affect consumers' defensive reaction tendencies and WSP. Second, as for the character of individuals, the locus of control can differ among individuals. The term *locus of control* refers to the degree to which an individual believes that he/she has control over the outcome of an event as opposed to external forces beyond his/her control (Rotter, 1966). More specifically, those with an internal locus of control tend to believe that certain events mainly depend on their behaviors and internal factors and that they have control over their lives. They are also more convinced that they can achieve positive results on their own. Thus, they are more inclined to take responsibility for change (April et al., 2012) and be more inclined to purchase products that can improve their shortcomings. Conversely, those with an external locus of control generally believe that certain events primarily depend on external factors such as luck and other influences. Since they also believe they do not have control over their lives, they are more inclined to blame others and the environment, rather than changing certain situations through their own efforts (Jacobs-Lawson et al., 2011). As a result, they are more likely to adopt defense mechanisms, with negative attitudes toward products claiming to improve their shortcomings. Third, as for the situational factors, individuals' perceived control can vary. In some situations, an individual can have a strong sense of control, whereas, in other situations, he/she can have a weak sense of control (Cutright and Samper, 2014). We also assert that such situational perceived control can also affect consumers' defensive reaction tendencies and WSP. Hence, the present study examines the role of these three sources of perceived control in three different experiments.

### Within-Domain and Without-Domain Improvement Products

There are many types of self-improvement products. Will consumers with low perceived control show a low WSP with all types of self-improvement products? We introduce product type as a moderator to explore this question. According to whether self-improvement products are related to consumers' current self-deficit, they can be divided into within-domain and without-domain improvement products. The former refers to products claiming to improve consumers' current self-deficit, and the latter refers to products claiming to improve a certain trait unrelated to consumers' current self-deficit (Kim and Gal, 2014). For example, when a consumer is feeling a deficiency in learning ability, a product claiming to improve consumers' learning ability is a typical within-domain improvement product, and a product claiming to improve consumers' physical appearance is a typical without-domain improvement product. Notably, the product type discussed in Hypotheses 1 and 2 is a within-domain improvement product. Whether perceived control has similar effects on without-domain improvement products has not been discussed. Based on the rationale in *Why Consumers Show a Low WSP in Self-deficit Situations*, the reason is that products claiming to improve their current deficiency sometimes function as a reminder of the self-deficit (Lisjak et al., 2015). Thus, consumers tend to avoid such products and seek other alternatives when they show defensive reaction tendencies toward their current self-deficits. Since without-domain improvement products are not related to consumers' current deficiencies, they need not show a defensive attitude to such products because they have not been threatened in these aspects. Based on this logic, the following hypothesis is posited:

**Hypothesis 3**: Product type moderates the effect of perceived control on WSP. More specifically, perceived control affects consumers' willingness to purchase within-domain improvement products under self-deficit situations but not without-domain improvement products.

We examined the above hypotheses by conducting three experiments. The experiments are organized as follows. First, Experiment 1 examines the impact of the controllability of self-deficits which is a source of perceived control on their WSP, and the mediating role of defensive reaction tendencies. Second, to enhance the robustness of this study, Experiment 2 focuses on another source of perceived control, i.e., the locus of control, and examines the effect of consumers' locus of control on their WSP as well as revalidates the mediating role of defensive reaction tendencies. Finally, since the controllability of self-deficits and consumers' locus of control is comparatively stable, Experiment 3 manipulates situational perceived control to determine its effect on consumers' WSP. It also investigates self-improvement products in more detail by dividing them into within-domain improvement products and without-domain improvement products.

## Experiment 1

The main objective of Experiment 1 was to verify H1 and H2 using the perceived controllability of consumers' self-deficit as the source of perceived control. In this case, intelligence was chosen as the element for the manipulation of self-deficit.

### Experimental Design and Participants

Experiment 1 adopted a 2 (intelligence deficit: deficit vs. non-deficit) × 2 (perceived controllability: high vs. low) between-subjects design. In addition, 140 undergraduates from a Chinese university were recruited. After excluding eight participants for failing to complete the experiment, 132 valid participants remained (*M*_age_ = 21.36, *SD*_age_ = 1.21), including 60 males and 72 females. Before the experiment, the participants were asked the following:

*Some people believe that intelligence is determined by uncontrollable factors, such as inheritance, development, and external environment, while others believe that intelligence is determined by controllable factors such as effort, training, and educational level. To what extent do you agree that an individual's intelligence is under his/her control? Regarding your response, “1” stands for “completely out of your control” and “7” stands for “completely within your control.”*

According to the average scores, the participants were assigned to either the high controllability group or the low controllability group. Then, they were randomly assigned to the intelligence deficit group and the non-intelligence deficit group.

### Experimental Procedure

This experiment was conducted in a laboratory with groups of participants who were told that they were about to complete multiple independent tasks. First, the intelligence deficit of the participants was manipulated by using the same method as Zhao et al. (2020). More specifically, the participants answered 12 questions from the online version of Raven's Progressive Matrices IQ Test. In order to create a lack of certainty regarding their answers to the questions, a 15-s time limit was imposed for each question. Consequently, the average accuracy rate was 61%. After the test, the participants in the intelligence deficit group were informed that according to the test results, their intelligence level ranked among the lowest 10% of the participants. Meanwhile, the participants in the non-intelligence deficit group were informed that according to the test results, their intelligence level ranked in the average range. In order to check the validity of the intelligence deficit manipulation, the participants were asked to rate the question “According to your test result, what is your intelligence level compared to the other participants?” based on a seven-point scale ranging from 1 (very low) to 7 (very high).

Second, according to the definition of self-improvement products (i.e., products that help consumers to compensate for their deficiencies by improving corresponding ability), books and courses are typical examples; therefore, studies have mainly used such products as their experimental material (e.g., Kim and Gal, 2014; Mandel et al., 2017; Zhao et al., 2020). Thus, we used such products as experimental material in our experiments. As for the measurement of the dependent variable, the participants have presented the book *Labyrinths of Reason*, and told that it could effectively improve their reasoning ability and intelligence. Then, in order to measure their WSP, they were asked to rate the following three statements based on a seven-point scale ranging from 1 (completely disagree) to 7 (completely agree): (1) I feel that I need this book at this time; (2) I want to purchase this book at the moment; and (3) This book is somewhat suitable for me. The average score of these three items was used as the index of WSP in this experiment (α = 0.81).

Third, as for the measurement of defense mechanisms, various tools in psychology studies exist such as Gleser and Ihilevich's (1969) Defense Mechanisms Inventory and Vaillant's (1976) Defense Mechanisms Rating Scale. However, these tools must be completed by professional psychological counselors, based on their inquiries and observations of the subjects. Moreover, although Bond et al. (1983) developed the Defense Style Questionnaire for self-assessment, this questionnaire primarily measures the personality traits of participants who use a particular defense mechanism over a long time. Thus, the aforementioned tools were not suitable for the context of this experiment.

To measure the defensive reaction tendencies of the participants in this experiment, three statements were compiled, following Ruttan and Nordgren's (2016) study on defensive information processing: (1) I do not believe that I have any intelligence deficits; (2) The information suggesting that I am intelligent deficient is not convincing; and (3) I do not believe that I need to compensate for my intelligence. The average score of these three statements was used as an indicator of defensive reaction tendencies (α = 0.87).

Finally, it is important to note that all of the materials in this study were in Chinese, including this manuscript, which was translated into English and edited by a native English copy editor. This study was approved by an ethics committee, and all of the participants attended the experiments voluntarily (with monetary payment) and were debriefed after each experiment.

### Results

#### Intelligence Deficit Manipulation Check

A 2 (intelligence deficit: deficit vs. non-deficit) × 2 (perceived controllability: high vs. low) analysis of variance (ANOVA) was performed on the participants' self-reported intelligence level. Based on the findings, there was a significant main effect regarding the intelligence deficit [*M*_deficit_ = 3.79 vs. *M*_non−deficit_ = 4.56; *F*_(1, 128)_ = 44.63, *p* = 0.000], whereas the main effects of perceived controllability and interaction were not significant (*Fs* ≤ 2.46, *ps* ≥ 0.120).

#### Control Variables

Since the gender of the participants and their economic conditions may have affected perceived controllability and WSP, respectively, this study analyzed these aspects as control variables. First, it conducted a logistic regression, with gender as the dependent variable (male = 0, female = 1). Moreover, the intelligence deficit (deficit = 0, non-deficit = 1), perceived controllability (low = 0, high = 1), and their interactive item served as independent variables. According to the findings, none of the main and interactive effects were significant [*Wald* (χ^2^) s ≤ 2.74, *p*s ≥ 0.098], suggesting that the participants' gender was balanced across the four experimental groups and did not confound the main results. Second, this study conducted a 2 (intelligence deficit: deficit vs. non-deficit) × 2 (perceived controllability: high vs. low) ANOVA on the participants' monthly consumption. The results showed that none of the main and interactive effects were significant (*F*s ≤ 0.22, *p*s ≥ 0.641), suggesting that the participants' economic conditions were balanced across the four experimental groups and did not confound the main results.

#### Defensive Reaction Tendencies

A 2 (intelligence deficit: deficit vs. non-deficit) × 2 (perceived controllability: high vs. low) ANOVA was performed on the participants' defensive reaction tendencies. According to the findings, there was a significant two-way intelligence deficit × perceived controllability interaction [*F*_(1, 128)_ = 4.54, *p* = 0.035, η2 p = 0.03], a significant main effect of the intelligence deficit [*F*_(1, 128)_ = 34.45, *p* = 0.000, η2 p = 0.21], and a significant main effect of perceived controllability [*F*_(1, 128)_ = 21.38, *p* = 0.000, η2 p = 0.14]. In addition, an analysis of simple effects showed that the participants in the low controllability group had significantly greater defensive reaction tendencies when receiving the intelligence deficit information, compared to receiving the non-intelligence deficit information [*M*_deficit_ = 5.17, *SD*_deficit_ = 1.10 vs. *M*_non−deficit_ = 3.81, *SD*_non−deficit_ = 0.56; *F*_(1, 128)_ = 30.18, *p* = 0.000, η2 p = 0.19]. Such a difference was also observed among the participants in the high controllability group, but with a lesser effect [*M*_deficit_ = 4.02, *SD*_deficit_ = 1.22 vs. *M*_non−deficit_ = 3.38, *SD*_non−deficit_ = 0.89; *F*_(1, 128)_ = 7.44, *p* = 0.007, η2 p = 0.06].

Overall, the findings indicate that when the participants were threatened by the self-deficit information, they exhibited more defensive reaction tendencies, regardless of whether they were in the high or low perceived controllability group. However, consistent with the aforementioned hypothesis, the participants in the low controllability group showed significantly more defensive reaction tendencies than those in the high controllability group when receiving the intelligence deficit information [*M*_low controllability_ = 5.17, *SD*_low controllability_ = 1.10 vs. *M*_high controllability_ = 4.02, *SD*_high controllability_ = 1.22; *F*_(1, 128)_ = 22.82, *p* < 0.000, η2 p = 0.15], whereas the effect was marginally significant when receiving the non-intelligence deficit information [*M*_low controllability_ = 3.81, *SD*_low controllability_ = 0.56 vs. *M*_high controllability_ = 3.38, *SD*_high controllability_ = 0.89; *F*_(1, 128)_ = 3.11, *p* = 0.080]. Moreover, the low perceived controllability aggravated the participants' defensive reaction tendencies, especially when they were threatened by the self-deficit information.

#### WSP

**Figure 1:**
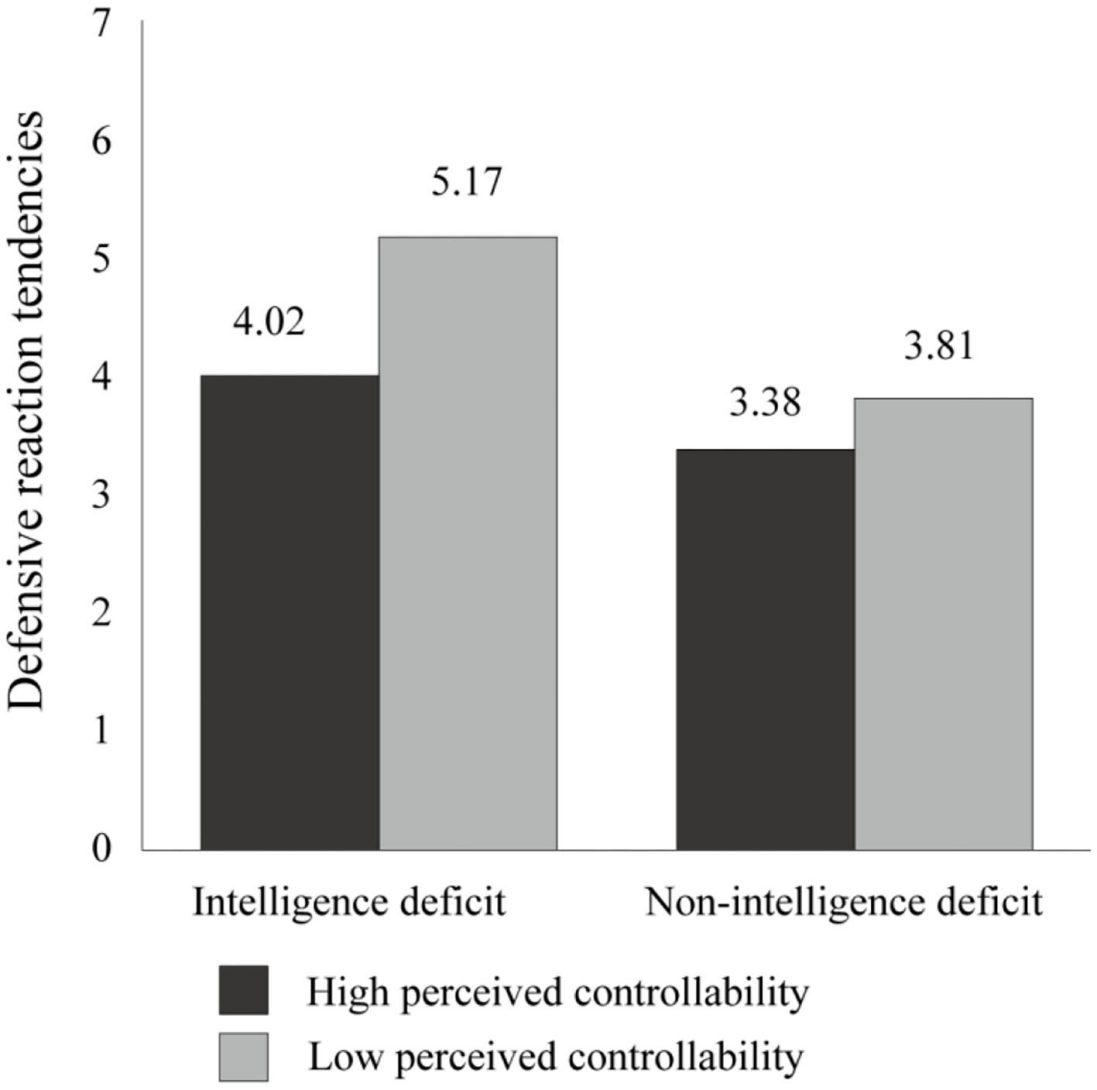
Results of Experiment 1: defensive reaction tendencies, as a function of intelligence deficit and perceived controllability.

**Figure 2:**
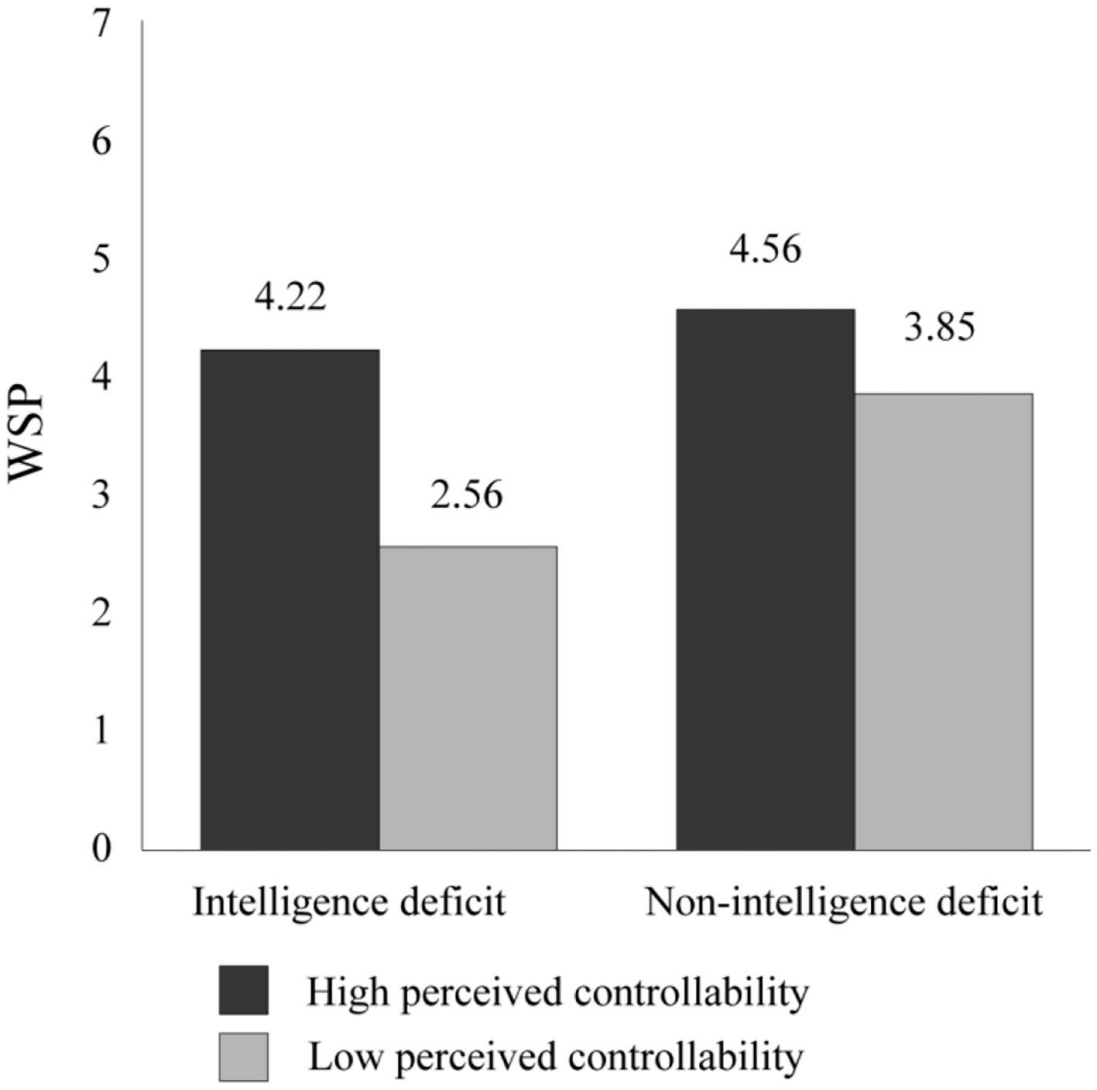
Results of Experiment 1: willingness to purchase self-improvement products (WSP) as a function of intelligence deficit and perceived controllability.

A 2 (intelligence deficit: deficit vs. non-deficit) × 2 (perceived controllability: high vs. low) ANOVA was conducted on the participants' WSP (Figure 1). Based on the results, there was a significant two-way interaction [*F*_(1, 128)_ = 5.17, *p* = 0.025, η2 p = 0.04], a significant main effect of the intelligence deficit [*F*_(1, 128)_ = 15.35, *p* = 0.000, η2 p = 0.11], and a significant main effect of perceived controllability [*F*_(1, 128)_ = 32.38, *p* = 0.000, η2 p = 0.20], suggesting that the participants in the high controllability group had significantly higher WSP (*M*_high controllability_ = 4.39, *SD*_high controllability_ = 1.28) than those in the low controllability group (*M*_low controllability_ = 3.20, *SD*_low controllability_ = 1.27) (Figure 2). In addition, an analysis of simple effects indicated that the participants in the low controllability group showed significantly less WSP for *Labyrinths of Reason* when receiving the intelligence deficit information than receiving the non-intelligence deficit information [*M*_deficit_ = 2.56, *SD*_deficit_ = 1.19 vs. *M*_non−deficit_ = 3.85, *SD*_non−deficit_ = 1.00; *F*_(1, 128)_ = 18.06, *p* = 0.000, η2 p = 0.12]. However, such a difference was not observed among the participants in the high controllability group [*M*_deficit_ = 4.22, *SD*_deficit_ = 1.30 vs. *M*_non−deficit_ = 4.56, *SD*_non−deficit_ = 1.25; *F*_(1, 130)_ = 1.44, *p* = 0.232]. This finding implies that the threat evoked by the intelligence deficit information can weaken the willingness to purchase intelligence-enhancing products among those who believe that their intelligence is uncontrollable, but not among those who believe that their intelligence is controllable. Consistent with the aforementioned hypothesis, the participants in the low controllability group showed significantly less willingness to purchase *Labyrinths of Reason* than those in the high controllability group when receiving the intelligence deficit information [*M*_low controllability_ = 2.56, *SD*_low controllability_ = 1.19 vs. *M*_high controllability_ = 4.22, *SD*_high controllability_ = 1.30; *F*_(1, 128)_ = 31.70, *p* = 0.000, η2 p = 0.20]. Moreover, a significant difference was found in the non-intelligence deficit condition, but with a lesser effect [*M*_low controllability_ = 3.85, *SD*_low controllability_ = 1.00 vs. *M*_high controllability_ = 4.56, *SD*_high controllability_ = 1.25; *F*_(1, 128)_ = 5.84, *p* = 0.000, η2 p = 0.04]. This finding implies that the participants who believe that their intelligence is uncontrollable have less willingness to purchase intelligence-enhancing products than those who believe that their intelligence is controllable and can be prompted by self-deficit information.

#### Mediating Role of Defensive Reaction Tendencies

**Figure 3:**
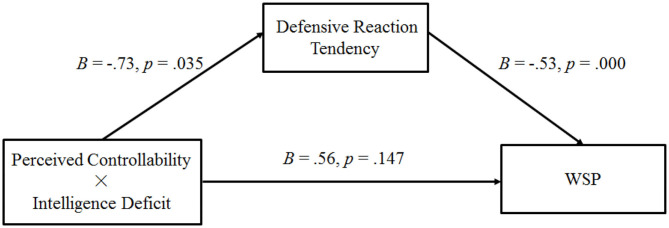
Results of Experiment 1: path analysis of Experiment 1 (WSP, willingness to purchase self-improvement products).

Using Model 8 of Hayes's (2013) process for testing moderated mediation, to examine Hypothesis 2, we employ the bootstrapping method (Preacher and Hayes, 2008). In this case, WSP served as the dependent variable, perceived controllability (low = 0, high = 1) served as the independent variable, defensive reaction tendencies were the mediator variables, and intelligence deficit was the moderator (non-deficit = 0, deficit = 1). Overall, the bootstrap analysis with 5,000 samples generated confidence intervals for the indexes of moderated mediation (the indirect effect of the highest-order interaction; 95% CI: [0.05, 0.83]). This result suggests that the interactive effect of perceived controllability and intelligence deficit on the participants' WSP was meditated by their defensive reaction tendencies (see Figure 3). Consistent with the aforementioned hypothesis, in the intelligence deficit condition, defensive reaction tendencies (95% CI: [0.30, 1.06]; indirect effect = 0.62) mediated the positive relationship between the participants' perceived controllability and WSP. In the non-intelligence deficit condition, the mediating effect was also significant (95% CI: [0.05, 0.47]; indirect effect = 0.23), but the effect was lesser than that of the intelligence deficit condition. This finding indicates that the participants' low perceived controllability of their intelligence evoked defensive reactions and weakened their WSP, while the intelligence deficit information further exacerbated this effect.

### Discussion

Based on the results, the self-threat evoked by the intelligence deficit information increased the participants' defensive reaction tendencies, especially those who believe that intelligence is uncontrollable and that perceived controllability can significantly reduce such tendencies. This suggests that enhancing the perceived controllability of individuals facing related deficits can effectively help eliminate their defensive reactions.

As for the analysis of WSP, it was found that the self-deficit information reduced the participants' WSP. However, this effect was not significant among those who believe that their deficit is controllable. Meanwhile, low perceived controllability significantly alleviated the participants' WSP, while high perceived controllability exacerbated it.

Finally, through the moderated mediation effect model, it was found that the participants' defensive reaction tendencies mediated the aforementioned process. Hence, Hypotheses 1 and 2 are verified. Moreover, perceived uncontrollability reduced the participants' WSP, even if they did not perceive themselves as deficient. Nevertheless, the effect of this situation was lesser than that of feeling deficient.

## Experiment 2

Experiment 1 validated the effect of the participants' perceived controllability of their deficits on WSP, and verified the mediating role of defensive reaction tendencies. However, the perceived control in Experiment 1 was primarily based on the participants' perceptions of whether their self-deficits were controllable. Since perceived can include multiple sources, it is unclear whether such sense from other sources will produce the same effect. Thus, Experiment 2, based on the personality traits of the participants, explored the influence of the locus of control (internal and external) on their WSP, and adopted the deficit of social relations as the method of manipulation. The main objective of Experiment 2 was also to verify H1 and H2.

### Experimental Design and Participants

Experiment 2 adopted a single-factor (social relations deficit: social exclusion, control group) between-subject design. In addition, 128 Chinese MBA students were recruited (*M*_age_ = 31.02, *SD*_age_ = 1.92), including 63 males and 65 females.

### Experimental Procedure

Overall, this experiment involved the manipulation of social exclusion to examine the social relations deficit. Before the experiment, the participants' internal and external control (α = 0.81) was measured with the Chinese version of the Internal-External Locus of Control Scale, originally compiled by Rotter (1966) and edited into Chinese by Wang (1991). The participants were then randomly assigned to either the experimental group or the control group, after which they performed an experience recall task that manipulated social exclusion (Chen et al., 2017). More specifically, in the experiment group, the participants were asked to recall an experience in which they were excluded by their friends, family, or someone they cared about. In the control group, the participants were asked to recall an experience at dinner. The participants in both groups were then asked to write (in detail) about their experiences and feelings in at least 100 words. To test the effect of the aforementioned manipulation method, the participants were asked to rate the following two questions on a five-point scale ranging from 1 (very low) to 5 (very high): (1) To what extent did you feel ignored during this experience?; and (2) To what degree did you feel rejected during this experience?

### Variable Measurement

In the measurement of the dependent variable, the book *How to Communicate Successfully in Any Situation* was selected. The premise of this book was that it could help readers improve their interpersonal skills and become more “popular.” After reading the introduction to the book, the participants were asked to rate the following three statements on a seven-point scale ranging from 1 (totally disagree) to 7 (totally agree): (1) I want to read this book now; (2) I think this book is exactly what I need; and (3) I am willing to purchase this book. The average score of the three statements was used as an indicator of WSP (α = 0.94).

Previous studies have shown that consumers using defense mechanisms tend to have more external attribution, rather than internal attribution to certain threats (Baumeister et al., 1998). Thus, Experiment 2 tested the participants' defensive reaction tendencies by asking them to rate the following three statements on a seven-point scale ranging from 1 (totally disagree) to 7 (totally agree): (1) Most of the time, I do not achieve the popularity that I deserve because other people are not friendly enough; (2) Making myself better is important for increasing my popularity; and (3) Most of the time, the reason why I am rejected or ignored by others has nothing to do with me. The average score of the three questions was used as an indicator of defensive reaction tendencies (α = 0.94). Finally, it is important to note that the materials, translation procedures, ethical issues, and the recruiting and debriefing procedures were the same as those in Experiment 1.

### Results

#### Manipulation Check

An independent sample *t*-test was performed by using the degree of feeling ignored/rejected as the dependent variable. The results showed that for all of the variables, the difference between the social exclusion group and the control group was significant (*ts* ≥ 11.21, *ps* ≤ 0.000), thus indicating the successful manipulation of social exclusion.

#### Control Variables

This experiment analyzed the participants' gender and monthly consumption, as control variables, in the same manner as that in Experiment 1. First, it conducted a logistic regression analysis, with gender as the dependent variable (male = 0, female = 1) and social relations deficit as the independent variable (control group = 0, social exclusion group = 1). According to the results, the effect was insignificant (*Wald*χ^2^ = 0.00, *p* = 1.00), suggesting that the gender was balanced across the two groups and did not confound the main results. Second, the result of the independent sample *t*-test showed that the difference in monthly consumption between the social exclusion group and the control group was not significant [*t*_(126)_ = 1.39, *p* = 0.167], suggesting that the participants' economic conditions were balanced across the four experimental groups and did not confound the main results.

#### Main Result

**Figure 4:**
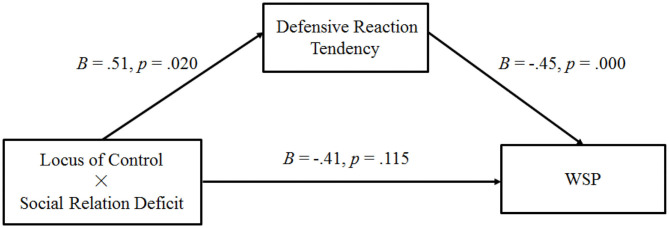
Results of Experiment 2: path analysis of Experiment 2 (WSP, willingness to purchase self-improvement products).

This experiment conducted a moderated mediation analysis by using the same bootstrapping method as that in Experiment 1. In this case, WSP served as the dependent variable, the locus of control served as the independent variable, defensive reaction tendencies were the mediator variables, and social relation deficit was the moderator (non-deficit = 0, deficit = 1). Overall, the bootstrap analysis with 5,000 samples generated confidence intervals for the indexes of moderated mediation (the indirect effect of the highest-order interaction; 95% CI: [−0.50, −0.05]). This finding indicates that the interactive effect of the locus of control and social relation deficit on the participants' WSP was mediated by their defensive reaction tendencies (see Figure 4).

Consistent with the aforementioned hypothesis, in the social relation deficit condition, defensive reaction tendencies (95% CI: [−0.53, −0.15]; indirect effect = −0.31) mediated the negative relationship between the locus of control and WSP. Moreover, since the participants with greater external control showed more defensive reaction tendencies toward the social relation deficit, their WSP was lower than those with greater internal control. Hence, Hypotheses 3 and 4 are verified. As for the non-social relation deficit condition, the mediating effect was not significant (95% CI: [−0.26, 0.06]; indirect effect = −0.08) (Table 1).

**Table 1:** Results of Experiment 2: indirect-direct effect of defensive reaction tendencies between the locus of control and willingness to purchase self-improvement products under social deficit and non-social deficit conditions.

	**Social relation deficit**			**Non-social relation deficit**		
	**Coefficient**	**LLCI**	**ULCI**	**Coefficient**	**LLCI**	**ULCI**
Indirect Effect	−0.31	−0.53	−0.16	−0.08	−0.28	0.06
Direct Effect	−0.39	−0.76	−0.02	0.02	−0.34	0.39

### Discussion

In contrast to the results of Experiment 1, the findings of Experiment 2 showed that the participants' WSP was not affected by the locus of control when they did not perceive their self-deficits. However, the effect was lesser than when they perceived their self-deficits. A possible reason for this is that when individuals believe that certain traits are uncontrollable, they do not believe the claims of certain self-improvement products, thus lowering their WSP.

## Experiment 3

In terms of the source of perceived control, the locus of control in Experiment 2 was primarily related to the personality traits of consumers, while the perceived controllability in Experiment 1 was mainly related to specific deficits. Thus, the following question is raised: Does more situational perceived control of consumers have the same effect? To answer this question, Experiment 3 manipulated the participants' situational perceived control and examined its effect on their WSP. In addition, Experiment 3 also examined the moderating role of product type which was proposed in H3. Thus, the main objective of Experiment 1 was to verify H1, H2 and H3.

### Experimental Design and Participants

Experiment 3 adopted a 2 (status deficit: deficit vs. non-deficit) × 2 (perceived control: high vs. low) × 2 (product type: within-domain vs. without-domain) between-subjects design. A total of 268 students from six MBA classes at two Chinese universities were recruited for this experiment (*M*_age_ = 31.45, *SD* = 3.71), including 152 males and 116 females who were randomly assigned to eight experimental groups.

### Experimental Procedure

This experiment used the status deficit as the manipulation method, following Zhao et al. (2018). First, a list of all of the students in each class was provided to each participant, after which he/she was asked to rate the status of each individual (including themselves) on a five-point scale ranging from 1 (representing the lowest 20% of the class) to 5 (representing the highest 20% of the class). After collecting the questionnaires, a statistical analysis of each individual's score was conducted. In this case, the average score given by the other participants constituted their status index in the class, thus reflecting their status in an objective manner. Second, a false status index was provided according to the experimental group to which they were randomly assigned. In this regard, the status deficit group was told that their status was judged by their classmates to be at the lowest level (the lowest 20% of the class), while the control group was told that their status was among the average level (the middle 20% of the class).

Before providing this false feedback, perceived control was manipulated with the method proposed by Cutright (2012). In this case, the participants in the high perceived control group were asked to recall and write (at least 100 words, in detail) about an event in which they had been threatened, but were able to control the outcome. Meanwhile, the participants in the low perceived control group were asked to recall and write (at least 100 words, in detail) about an event in which they had been threatened, but were unable to control the outcome.

To determine the effectiveness of this manipulation method, two post-graduate students were asked to independently rate the degree of perceived control on a seven-point scale ranging from 1 (representing extremely low perceived control) to 7 (representing extremely high perceived control). This was based on the participants' descriptions after the task was completed. The scores given by these two students were then totaled as the index of perceived control manipulation (*r* = 0.80). After obtaining this index, the false feedback was presented to the participants. Moreover, to test the status deficit manipulation, the participants were asked to rate their own status in the class, based on the feedback from their classmates. In this regard, their ratings ranged from 1 (extremely low status) to 7 (extremely high status).

### Variable Measurement

In the measurement of the dependent variables, one product was provided for the within-domain improvement product group, and one product was provided for the without-domain improvement product group. To select the two products more accurately, in the pilot study, 63 participants were chosen from the same sample for the main study and asked to rate the influence of individuals' leadership on their status within the group as well as the influence of individuals' creativity on their status within the group on a seven-point scale ranging from 1 (representing minimum influence) to 7 (representing maximum influence). Based on the findings, the participants believed that leadership had significantly more influence on their status in the group than creativity [*t*_(62)_ = 9.97, *p* = 0.000]. Thus, two products were simulated.

As for the product that claimed to improve status deficit, an MBA course called *Leadership Enhancement* was introduced to the participants in a written text, which aimed to cultivate their leadership skills and improve their status within an organization. For the product that claimed to improve other traits, another MBA course called *Creativity Enhancement* was introduced to the participants in a written text, which aimed to improve their creativity and make them more innovative in their work and studies. The participants were then asked to rate the following three items on a seven-point scale ranging from 1 (totally disagree) to 7 (totally agree): (1) I am willing to purchase this course; (2) I believe that I need this course; and (3) This course suits me. The average score of these three items was used as the index for WSP (α= 0.96). It is important to note that when measuring the defensive reaction tendencies, this experiment adopted the three items from Experiment 1, but replaced the intelligence item with status. The average score of the three items was used as the index for defensive reaction tendencies (α = 0.86). Again, the materials, translation procedures, ethical issues, and the recruiting and debriefing procedures were the same as those in Experiment 1.

### Results

#### Status Deficit Manipulation Check

A 2 (status deficit: deficit vs. non-deficit) × 2 (perceived control: high vs. low) × 2 (product: within-domain vs. without-domain) ANOVA was performed on the self-reported status of the participants. Overall, there was a significant main effect of status deficit [*M*_deficit_= 5.92 vs. *M*_non−deficit_= 3.32; *F*_(1, 260)_ = 1463.02, *p* = 0.000], whereas the other main effects and interactions were not significant (*Fs* ≤ 1.212, *ps* ≥ 0.272).

#### Perceived Control Manipulation Check

A 2 (status deficit: deficit vs. non-deficit) × 2 (perceived control: high vs. low) × 2 (product: within-domain vs. without-domain) ANOVA was performed on the coded control reflected in the participants' essays. Based on the findings, there was a significant main effect of perceived control [*M*_high_= 5.32 vs. *M*_low_= 2.71; *F*_(1, 260)_ = 1202.97, *p* = 0.000], whereas the other main effects and interactions were not significant (*Fs* ≤ 1.96, *ps* ≥ 0.163).

#### Control Variables

This experiment conducted the same control variable analysis as Experiments 1 and 2. According to the results, the participants' gender and their monthly consumption were balanced across groups and did not confound the main results.

#### WSP

A 2 (status deficit: deficit vs. non-deficit) × 2 (perceived control: high vs. low) × 2 (product type: within-domain vs. without-domain) ANOVA on the participants' WSP revealed a significant three-way interaction [*F*_(1, 260)_ = 18.28, *p* < 0.000, η2 p = 0.07]. In addition, a separate 2 (status deficit: deficit vs. non-deficit) × 2 (control: high vs. low) ANOVA for the within-domain improvement product revealed a significant two-way interaction [*F*_(1, 130)_ = 14.45, *p* < 0.000, η2 p = 0.10], while the same two-way ANOVA for the without-domain improvement product also revealed a significant two-way interaction [*F*_(1, 130)_ = 4.36, *p* = 0.039, η2 p = 0.03]. The simple effects for each product are reported as follows.

#### Within-Domain Improvement Product: Leadership Enhancement Course

**Figure 5:**
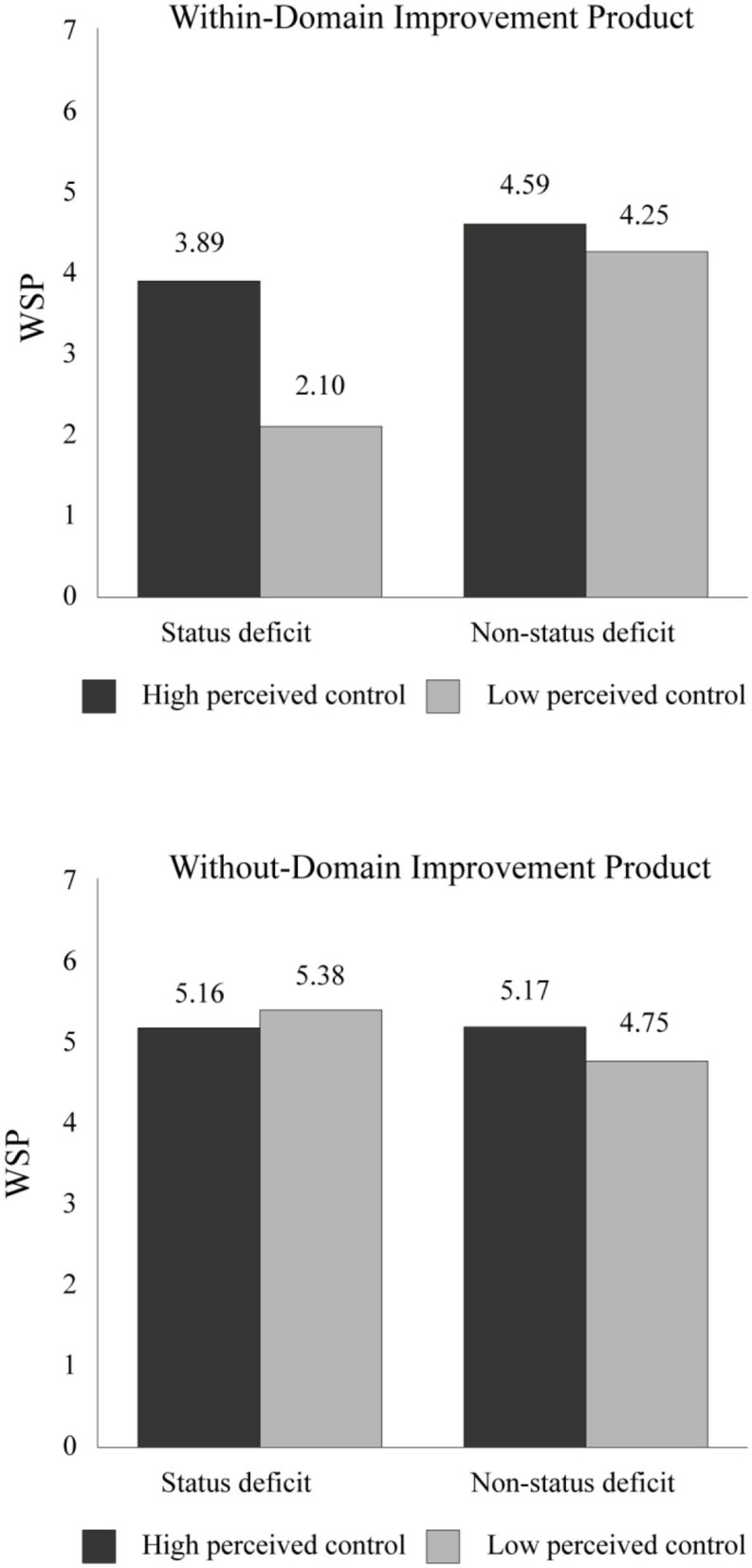
Results of Experiment 3: willingness to purchase self-improvement products (WSP) as a function of status deficit, perceived control, and product type.

The participants in the low control group showed significantly lower WSP for the *Leadership Enhancement Course* when receiving the status deficit information than when receiving the non-status deficit information [*M*_deficit_ = 2.10, *SD*_deficit_ = 1.08 vs. *M*_non−deficit_ = 4.25, *SD*_non−deficit_ = 0.78; *F*_(1, 130)_ = 64.59, *p* = 0.000, η2 p = 0.33]. Such a difference was also observed among the participants in the high control group, but with a lesser effect [*M*_deficit_ = 3.89, *SD*_deficit_ = 1.40 vs. *M*_non−deficit_ = 4.59, *SD*_non−deficit_ = 1.10; *F*_(1, 130)_ = 6.65, *p* = 0.011, η2 p = 0.05]. This finding indicates that when the participants were threatened by the self-deficit information, they showed a low willingness to purchase within-domain improvement products, with low perceived control exacerbating this tendency (see Figure 5). Moreover, consistent with the aforementioned hypothesis, the participants in the low control group showed significantly lower WSP for the *Leadership Enhancement Course* than those in the high control group when receiving the status deficit information [*M*_low control_ = 2.10, *SD*_low control_ = 1.08 vs. *M*_high control_ = 3.89, *SD*_high control_ = 1.40; *F*_(1, 130)_ = 43.01, *p* = 0.000, η2 p = 0.25]. However, the effect was not significant when receiving the non-status deficit information [*M*_low control_ = 4.25, *SD*_low control_ = 0.78 vs. *M*_high control_ = 4.59, *SD*_high control_ = 1.10; *F*_(1, 130)_ = 1.54, *p* = 0.216]. This finding shows that low perceived control only affected the participants' WSP when they felt threatened by the self-deficit information (see Figure 5).

#### Without-Domain Improvement Product: Creativity Enhancement Course

The participants in the low control group showed significantly higher WSP for the *Creativity Enhancement Course* when receiving the status deficit information than receiving the non-status deficit information [*M*_deficit_ = 5.38, *SD*_deficit_ = 0.68 vs. *M*_non−deficit_ = 4.75, *SD*_non−deficit_ = 1.21; *F*_(1, 130)_ = 8.58, *p* = 0.004, η2 p = 0.06]. However, such a difference was not observed among the participants in the high control group [*M*_deficit_ = 5.16, *SD*_deficit_ = 0.78 vs. *M*_non−deficit_ = 5.17, *SD*_non−deficit_ = 0.74; *F*_(1, 130)_ = 0.001, *p* = 0.981]. This finding implies that the self-deficit information increased the low perceived control participants and their willingness to purchase without-domain improvement products, compared to the high perceived control participants (see Figure 5). Moreover, it is possible to conclude that the self-deficit information did not evoke the participants' defensive attitudes toward without-domain improvement products, but prompted the low perceived control participants' willingness to purchase them.

Conversely, the low and high perceived control participants did not show a significant difference in WSP for the *Creativity Enhancement Course* when receiving the status deficit information [*M*_low control_ = 5.38, *SD*_low control_ = 0.68 vs. *M*_high control_ = 5.16, *SD*_high control_ = 0.78; *F*_(1, 130)_ = 1.06, *p* = 0.306]. Interestingly, the participants in the high perceived control group showed marginally more significant WSP toward the *Creativity Enhancement Course* than those in the low perceived control group when receiving the non-status deficit information [*M*_low control_= 4.75, *SD*_low control_ = 1.21 vs. *M*_high control_ = 5.17, *SD*_high control_ = 0.74; *F*_(1, 130)_ = 3.73, *p* = 0.056]. These findings suggest that high perceived control may increase consumers' willingness to purchase without-domain improvement products under non-threatening conditions (see Figure 5).

#### The Mediating Role of Defensive Reaction Tendencies

**Figure 6:**
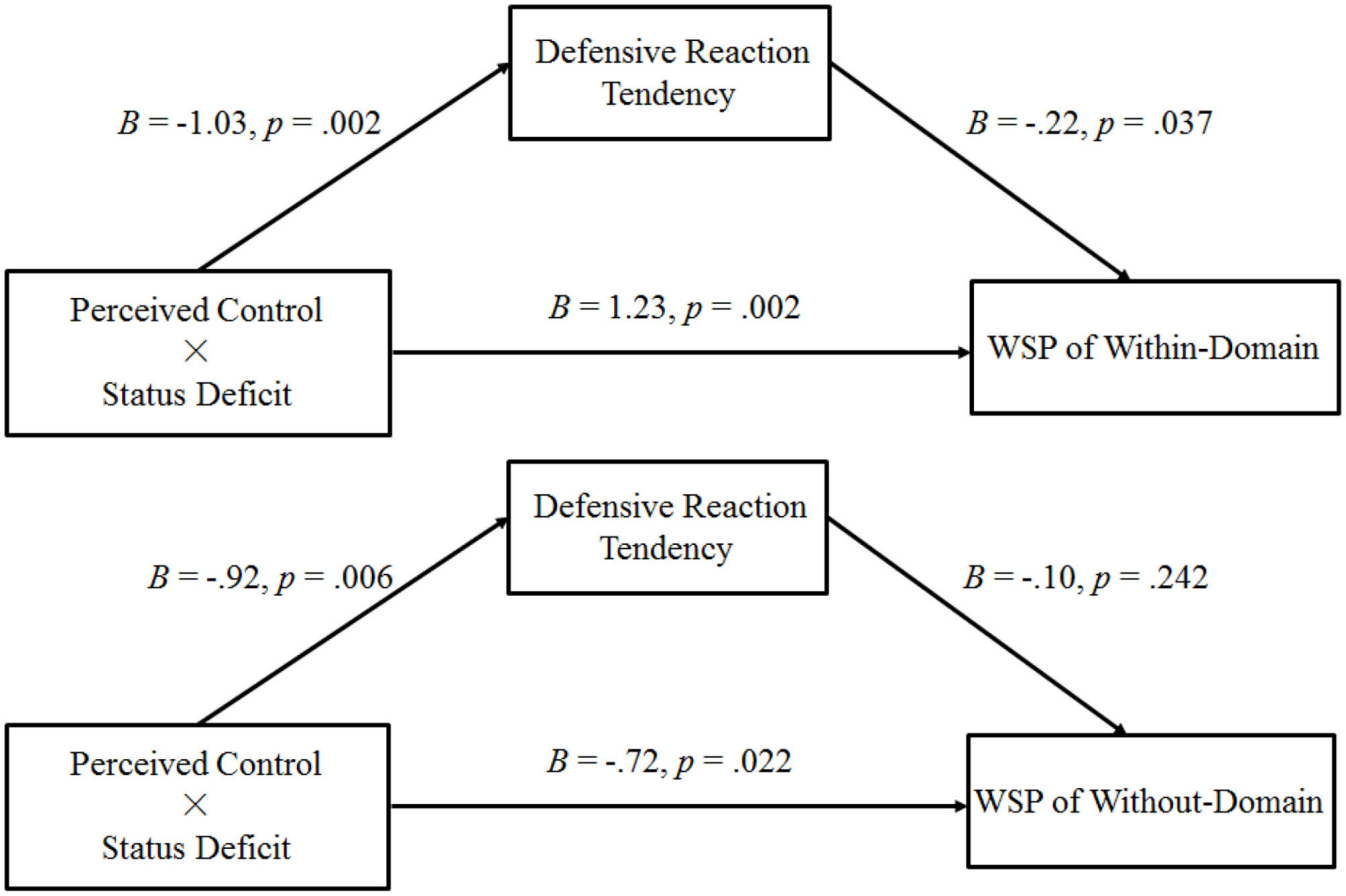
Results of Experiment 3: path analysis of Experiment 3 (WSP, willingness to purchase self-improvement products).

This experiment conducted a moderated mediation analysis by using the same bootstrapping method as that in previous experiments. In this case, WSP for the within-domain improvement product served as the dependent variable, perceived control was the independent variable (0 = low, 1 = high), defensive reaction tendencies were the mediator variables, and the status deficit was the moderator (0 = non-deficit, 1 = deficit). Overall, the bootstrap analysis with 5,000 samples generated confidence intervals for the indexes of moderated mediation (the indirect effect of the highest-order interaction; 95% CI: [0.03, 0.56]). This finding suggests that the interactive effect of perceived control and status deficit on the WSP of within-domain improvement goods was meditated by defensive reaction tendencies (see Figure 6). Consistent with the aforementioned hypothesis, in the status deficit condition, defensive reaction tendencies (95% CI: [0.03, 0.50]) mediated the positive relationship between perceived control and WSP for the within-domain improvement product. However, in the non-status deficit condition, the mediating effect was not significant (95% CI: [−0.14, 0.10]). This study also conducted the same moderated mediation analysis on WSP for the without-domain improvement product (see Figure 6), but the effect was not significant (the indirect effect of the highest-order interaction; 95% CI: [−0.05, 0.32]).

### Discussion

Experiment 3 manipulated the situational perceived control of the participants by asking them to recall a previous experience of high or low perceived control. It found that the perceived control of the participants affected their willingness to purchase within-domain improvement products. In particular, the participants with low perceived control showed stronger defensive reactions than those with high perceived control. Thus, they had a low willingness to purchase within-domain improvement products. This again verified the results of Experiments 1 and 2.

Experiment 3 also examined the effect of perceived control on without-domain improvement products. It found that the role of perceived control in the participants' willingness to purchase without-domain improvement products and within-domain improvement products was very different. More specifically, for the participants with low perceived control who were threatened by the self-deficit information, their willingness to purchase without-domain improvement products was even stronger than when they received the non-self-deficit information. This was the opposite tendency toward within-domain improvement products. This finding indicates that within-domain improvement products and without-domain improvement products can have different psychological compensation effects when consumers perceive their self-deficits.

Although the without-domain improvement products in Experiment 3 were designed as a control group for within-domain improvement products, they also verified that the effect of low perceived control on the participants' WSP was only reflected in the products that could improve their corresponding deficits, rather than all of the self-improvement products. Unexpectedly, this experiment found that the deficits enhanced the low perceived control participants to purchase without-domain improvement products. Such a phenomenon could be explained (to some extent) through the self-affirmation theory and the concept of fluid compensation (Mandel et al., 2017). In this case, self-affirmation refers to individuals affirming their self-worth in domains unrelated to current threats to maintain self-integrity, while fluid compensation refers to individuals gaining affirmation through other traits to compensate for certain threats caused by their deficiencies. These findings imply that when consumers with low perceived control have low WSP toward within-domain improvement products, without-domain improvement products might make them feel that they can compensate for their existing inadequacies from other perspectives. Thus, they might prefer fluid compensation through without-domain improvement products. However, future studies should investigate this hypothesis.

## General Discussion

Based on the theory of psychological defense mechanisms, consumers with low perceived control will show stronger defensive reaction tendencies when facing a psychological threat caused by their self-deficits, thus lowering their willingness to purchase the corresponding self-improvement products. Similarly, the three experiments in the present study found the following: (1) Consumers who feel that their deficits are uncontrollable are more likely to show a lower WSP than those who feel that their deficits are controllable; (2) Consumers with an external locus of control tend to show a lower WSP than those with an internal locus of control when facing their inadequacies; and (3) Consumers with lower perceived control generally show a lower WSP than those with higher perceived control.

### Theoretical Contributions

Previous literature has focused on consumers' self-compensation by purchasing self-improvement products (Kim and Rucker, 2012), with less attention on when consumers' willingness to purchase these products decreases. In addition, the literature on how consumers deal with psychological threats caused by their deficits has mainly concentrated on the symbolic self-completion and self-affirmation theories. In this regard, the symbolic self-completion theory focuses on consumers' compensating behaviors through products that can symbolize their “excellence” in domains in which they feel threatened. For example, when individuals feel that they lack status or power, they may purchase products with a symbolic meaning to restore their sense of status and power (Rucker and Galinsky, 2008, 2009; Dubois et al., 2012). As for the self-affirmation theory, it emphasizes consumers' behavior of seeking advantages in other domains to compensate for their deficiencies in a certain domain. For example, Sobol and Darke (2014) found that consumers may compensate for a lack of physical attractiveness by highlighting their intellectual advantages, while Duclos et al. (2013) found that individuals will compensate for deficits in social relations through the pursuit of money. However, the focus of the aforementioned studies was primarily on how individuals' deficits influence their consumption, with less attention on how these deficits inhibit their consumption of specific products.

Defense mechanisms have been extensively studied in the field of social psychology, but these studies have mainly focused on how individuals' defense mechanisms affect their stress management, mental health, and social behavior (Inzlicht and Kang, 2010; Stroebe and Schut, 2010). Meanwhile, the research on how these mechanisms affect individuals' consumption behavior is limited. Thus, by introducing the theory of psychological defense mechanisms, the present study explained the psychology behind consumers' willingness (or unwillingness) to purchase products that can improve their deficits. It is hoped that the findings will help expand the theoretical basis and research issues in related fields.

Based on the theory of psychological defense mechanisms, this study found that perceived control is an important factor affecting consumers' WSP. Existing research on the factors that influence consumer behaviors in the context of self-threats has mainly focused on when the threat occurs, the type of threat, and the individuals' self-regulating strategies in response to the threat. For example, Kim and Rucker (2012) found that when consumers are conducting proactive consumption, due to a possible threat, they are more likely to choose products that are related to the threatened trait. However, when consumers are demonstrating reactive consumption in response to a threat, they are more likely to choose products that are not related to the threatened trait. In related studies, Kim and Gal (2014) found that when consumers feel threatened, they will respond with compensatory consumption. If they are guided to self-acceptance, they will have adaptive consumption. With regard to certain types of threats, Han et al. (2015) found that intellectual threats and mortality salient threats can trigger approach motivations and problem-focused coping strategies, while other types of threats, such as social exclusion and personal control threats, can trigger avoidance motivation and emotion-focused coping strategies.

In the present study, three sources of perceived control, i.e., situational perceived control, perceived controllability of threat, and internal-external locus of control, were chosen to explore their influence on consumers' WSP. This research not only focused on the aforementioned multiple sources of control to expand the theoretical perspectives of perceived control theory, but it also provided suggestions and guidance for practice. The existing literature on perceived control has generally focused on how the lack of perceived control is embodied in consumers' consumption of specific products. For example, consumers with low perceived control may prefer “lucky” products (i.e., those associated with positive outcomes); products that require more effort; and logos and products with boundaries (Cutright, 2012; Hamerman and Johar, 2013; Cutright and Samper, 2014). However, these studies have ignored whether the lack of perceived control will weaken an individual's tendency to consume certain types of products. Therefore, the results of this study also provide new research ideas and inspiration for fields related to perceived control.

Finally, this study also contributes to the literature by introducing product type as a moderator. We found that the effect of consumers' perceived control on WSP is different between within-domain and without-domain products and the underlying mechanism. Specifically, perceived control affects consumers' willingness to purchase within-domain improvement products under self-deficit situations but not without-domain improvement products. Defense mechanism theory explains the underlying mechanism between perceived control and consumers' WSP for within-domain improvement products but not without-domain improvement products. This finding clarified the boundary conditions of the present study. It could also be used to explore what factors affect consumers' WSP for without-domain improvement products and their underlying mechanism. We plan to explore this topic in future research.

### Practical Implications

The results of this study will be conducive to the application and practice of business strategies. In daily life, consumers often come across various cues that imply that they are deficient in some aspect. Moreover, many brands depend on the use of psychological threats to consumers' self-image as one of their marketing strategies. However, regardless of whether businesses are targeting consumers' feelings of inadequacy or posing such threats to consumers unintentionally, understanding the types of situations in which consumers show a low willingness to purchase will benefit their financial goals. For example, as shown in the present study, in the face of a psychological threat, the participants with high perceived control accepted their recommendations and purchased products that promised self-improvement. However, the participants with low perceived control adopted defensive mechanisms and had low WSP. Based on this finding, businesses must help consumers restore their perceived control over their deficits while presenting their perceptions of their deficiencies.

This study examined the effect of three sources of perceived control; according to the findings, it is possible to enhance consumers' perceived control over their deficits in the following aspects. First, all issues are not controllable or uncontrollable. By advertising that the trait improved by the product is controllable, consumers' perceptions that their deficits are uncontrollable can be reversed, which, in turn, will help restore their perceived control and increase their WSP. Second, helping consumers realize that their deficits are attributed to internal factors, rather than external factors, can also restore their perceived control. For example, the slogan “Your destiny is in your hands” encourages a consumer to see an internal locus of control. It is also possible to inspire consumers to perceive an internal locus of control over their own destiny and assume responsibility for it by creating a responsible, positive, and courageous brand image. On the other hand, businesses may choose a self-reliant and well-respected public figure as the spokesperson of their brand to promote the belief that people can control their own destiny and stimulate the perceived control of consumers. Notably, the results of an empirical study indicate that the aforementioned practical implications are effective only for those who sell within-domain improvement products.

### Limitations and Future Directions

In this study, different types of deficits were manipulated through three experiments, and different types of experimental materials and products were selected to verify the effect of perceived control on consumers' WSP, all of which increased the internal validity and robustness of the results. However, there are several limitations worth noting. First, the three experiments in this study were all carried out in laboratories. Although the control over internal validity was good, the external validity was relatively weak because there was no field experiment. Thus, future research should conduct field experiments in a real environment to increase the external validity of the results. Second, although this study chose representative self-improvement products that claimed to improve different domains in the three experiments, they were somewhat similar. Hence, future studies should include other types of products to generalize the findings. Third, since the self-deficits and perceived control in this study were mainly induced by experimental manipulation, it is unclear whether the long-term deficits or perceived control of consumers could affect their WSP. Thus, future research should complement the aforementioned problems with a combination of research methods such as questionnaires and qualitative research. Finally, although this study found that the participants' WSP was low when they had low perceived control, it is still unclear whether such control can promote the purchase of other types of products such as hedonic or conspicuous ones. Therefore, future studies should explore whether the tendencies of consumers to purchase other types of products are affected, especially as their WSP decreases.

## Data Availability Statement

The raw data supporting the conclusions of this article will be made available by the authors, without undue reservation.

## Ethics Statement

The studies involving human participants were reviewed and approved by the School of Philosophy and Sociology, Jilin University, China. Written informed consent from the participants' was not required to participate in this study in accordance with the national legislation and the institutional requirements.

## Author Contributions

All authors listed have made a substantial, direct and intellectual contribution to the work, and approved it for publication.

## Conflict of Interest

The authors declare that the research was conducted in the absence of any commercial or financial relationships that could be construed as a potential conflict of interest.
